# Metabolic and molecular effects of dietary extra virgin olive oil in blood and placenta of women with GDM

**DOI:** 10.3389/fendo.2023.1219276

**Published:** 2023-08-15

**Authors:** Dalmiro Gomez Ribot, Esteban Diaz, María Victoria Fazio, Hebe Lorena Gómez, Valeria Careaga, Marta Maier, Silvia Beatriz Macchi, Carlos Alberto Gresta, Evangelina Capobianco, Alicia Jawerbaum

**Affiliations:** ^1^ Facultad de Medicina, Universidad de Buenos Aires (UBA), Buenos Aires, Argentina; ^2^ Consejo Nacional de Investigaciones Científicas y Técnicas (CONICET) – Universidad de Buenos Aires (UBA), Laboratory of Reproduction and Metabolism, Centro de Estudios Farmacológicos y Botánicos (CEFYBO), Buenos Aires, Argentina; ^3^ Department of Obstetrics, Hospital General de Agudos Dr. Ignacio Pirovano, Buenos Aires, Argentina; ^4^ Unidad de Microanálisis y Métodos Físicos Aplicados a Química Orgánica (UMYMFOR) [Consejo Nacional de Investigaciones Científicas y Técnicas (CONICET)-Universidad de Buenos Aires (UBA)], Department of Organic Chemistry, School of Exact and Natural Sciences, University of Buenos Aires, Buenos Aires, Argentina

**Keywords:** maternal diabetes, extra virgin olive oil, placenta, metabolism, MiR-199

## Abstract

Gestational diabetes mellitus (GDM) increases the risks of maternal, placental, and neonatal complications. Previously, we found that a diet enriched in extra virgin olive oil (EVOO) prevents increased maternal triglyceridemia and placental proinflammatory markers in a cohort of GDM patients. The aim of this work was to evaluate maternal circulating markers of insulin resistance, placental collagen, glycogen and lipid levels, and placental levels of proteins, mRNAs, and a microRNA involved in the endocytic pathway in the same cohort of control women and women with GDM who received or did not receive a diet enriched in EVOO (36 g/day) from weeks 24 to 28 of pregnancy until term.

**Results:**

At term, the TG/HDL cholesterol ratio, fatty acid binding protein 4 circulating levels, and maternal BMI were increased in the GDM patients, alterations prevented by the maternal diet enriched in EVOO. Although there were no changes in placental lipid levels and lipid profile, GDM placentas were thicker than controls and showed increased glycogen and collagen content, alterations prevented by the EVOO enriched diet. GDM placentas showed increases in megalin levels, in the expression of several genes involved in the endocytic pathway, and in miR-199, which targets these genes, alterations prevented by the maternal diet enriched in EVOO.

**Conclusions:**

We identified novel beneficial effects of an EVOO-enriched diet in GDM women, a diet capable of regulating maternal insulin resistance, the structure and metabolism of the placenta, and the placental endocytic pathway, suggesting effects that may be beneficial for fetal development.

## 1 Introduction

Gestational diabetes mellitus (GDM) is a prevalent disease worldwide, affecting 1 to 6–10 pregnant women in many developed, underdeveloped, and developing countries (1, 2). GDM patients are at increased risks for maternal and neonatal complications. Maternal complications include increased risks for preeclampsia, cardiovascular diseases, cesarean sections in the short term, and increased risk of type 2 diabetes in the long term (3). Neonatal complications include increased risks for macrosomia, respiratory distress syndrome, neonatal hypoglycemia, and fetal programming of metabolic and cardiovascular diseases (4, 5). The placenta plays a crucial role in fetal programming, and its impairments can predict offspring’s long-term health (6). Previous studies have shown alterations in morphology, histology, and function in GDM placentas (7, 8). In GDM, the placenta is both exposed to and a generator of prooxidant and proinflammatory molecules (9). The prooxidant and proinflammatory intrauterine environment affects the crucial role of the placenta in the regulation of the multiple aspects needed for proper maternal adaptation and proper fetal development and nutrition (10). A good metabolic control is a key factor in the prevention of both maternal and neonatal short- and long-term complications, but, even with a good metabolic control, complications may remain (11).

Nutrigenomic agents are increasingly recognized as valuable tools to prevent different diseases (12). Extra virgin olive oil (EVOO) is a nutrigenomic agent that has been shown to prevent metabolic, cardiovascular, and proinflammatory diseases out of pregnancy (13–15). In pregnancy, the capacity of EVOO to prevent the induction of GDM has been reported (16–18). EVOO is enriched in polyphenols, which possess well-known antioxidant properties, and in oleic acid, a monounsaturated fatty acid that can activate nuclear receptors, PPARs, master regulators of metabolic, anti-inflammatory, and developmental pathways (19–21).

Recently, we performed a clinical study addressing the effects of a diet supplemented with EVOO in GDM patients receiving (*n* = 15) or not receiving (*n* = 15) 36 g of EVOO daily. In that study, we found that well-controlled GDM patients from the non-supplemented group showed increased maternal triglyceridemia and increased proinflammatory markers in the placenta, alterations associated with impaired PPAR signaling and prevented by the maternal diet enriched in EVOO (22).

In metabolic diseases, both increased triglyceridemia and increased proinflammatory state are related to an increase in insulin resistance (23). Insulin resistance is part of the physiological adaptations to pregnancy, but increased insulin resistance is a hallmark in GDM, likely associated with increased circulating proinflammatory markers (10, 24). Among these markers, an increase in matrix metalloproteinases 2 and 9 (MMP2 and MMP9), proteolytic enzymes relevant for embryo and placental development, is associated with a proinflammatory state (9, 25). Previous studies in experimental models of diabetes have shown increased MMP9 and/or MMP2 in the placenta, fetuses, and maternal plasma (26). These studies have also shown that diets enriched in unsaturated fatty acids, capable of activating PPAR pathways, can prevent this overactivity (26, 27).

Lipid metabolic pathways, highly related to PPAR signaling and reflecting maternal metabolic impairments, can also be impaired in experimental models and patients with diabetes and pregnancy (28–30). In the placenta, collagen deposition, a marker of fibrosis, and accumulation of lipids or glycogen are alterations that have been found in the placenta of GDM patients (29, 31, 32). Through the placenta, free fatty acids are released from lipoproteins to be transferred to the fetus through receptor-mediated pathways, or transferred as lipoproteins through endocytic pathways (29, 33). In order to transfer lipoproteins through the endocytic pathway, the placenta expresses main proteins involved in lipoprotein endocytosis, such as low-density lipoprotein receptor (LDLR), low-density lipoprotein receptor-related protein 1 (LRP1, also known as apolipoprotein E receptor), low-density lipoprotein receptor-related protein 2 (LRP2, also known as megalin), cubilin, and clathrin (33–36). Most of the genes codifying for these proteins are negatively regulated by hsa-miR-199-5p (hereinafter miR-199), a microRNA involved in the regulation of lipoprotein endocytosis (37).

The present work is a secondary analysis performed in maternal blood and placentas in a cohort of control women and GDM women who received or did not receive a diet daily supplemented with 36 g of EVOO from weeks 24 to 28 of pregnancy until delivery. The aim of this work was to study markers of insulin resistance and MMP2 and MMP9 activities in maternal plasma, as well as to evaluate placental morphology, placental levels of collagen, glycogen and lipids, placental expression of proteins and genes involved in the endocytic pathways, and miR-199 levels in the placentas from both control patients and GDM patients treated or not with a diet enriched in three tablespoons of EVOO daily.

## 2 Materials and methods

### 2.1 Study design, dietary treatment, and tissue collection

The present study is a secondary analysis of a previous randomized trial in which control (*n* = 15) and GDM patients with a singleton fetus (*n* = 30) were enrolled between weeks 24 and 28 of pregnancy and either treated (*n* = 15) or not (*n* = 15) with a diet enriched in three tablespoons of crude EVOO daily (36 g/day) from enrollment and until term (22). The present work evaluated in the same cohort circulating insulin resistance markers and MMP activity as well as placental morphology, storage metabolites and proteins, and regulators of the placental endocytic pathway.

The patients were enrolled at Hospital Ignacio Pirovano, Buenos Aires, Argentina. GDM was diagnosed according to the diagnostic criteria of Latin American Diabetes Association (ALAD)/Argentine Society of Diabetes (SAD) (glycemia values >99 mg/dl in two measurements or glycemia values >140 mg/dl at 2 h after a universal p75g oral glucose tolerance test) (38). The study was approved by the Ethics and Research Committee of the Pirovano Hospital (Review Board Project: DI-2016-29-HGAIP) and registered at the Ministry of Health of the City of Buenos Aires, IF-2016-22533767. All participants provided written informed consent. Exclusion criteria included body mass index (BMI) over 30 kg/m^2^ and concurrent pathologies. Detailed exclusion criteria, design, adherence to the dietary treatment, and outcomes of the randomized trial have been previously reported (22).

Maternal blood samples were collected at enrollment and at term, whereas both blood samples and placentas were collected at term. Immediately after delivery, the umbilical cord was rapidly clamped, and the placenta carefully cleaned up from excess blood (39). Placental diameter and perimeter were measured with adequate measuring tools. Placental thickness at the center of the chorionic plate was measured by piercing the disc with a knitting needle in which millimeter marks were inscribed (40). Then, placental tissues from central cotyledons were obtained and appropriately stored as previously described for further analysis (22).

### 2.2 Metabolic parameters, placental morphometric analysis, and sample preparations

To study the ratio between circulating triglycerides (TG) and HDL cholesterol as a marker of insulin resistance (41), TG and HDL cholesterol levels were evaluated by colorimetric methods (Wiener lab, Rosario, Argentina) in 8-h fasting plasma at enrollment (gestational weeks 24–28) and at term (gestational week 37). Plasma was conserved at −80°C for further evaluation of fatty acid binding protein 4 (FABP4) [an insulin resistance marker (24)] and MMP2 and MMP9 activities [remodeling enzymes and markers of the proinflammatory state (25)]. BMI, defined as weight in kilograms divided by the square of the height in meters, was evaluated at enrollment and at term. At delivery, placental diameter, thickness, and perimeter were measured, and the placentas were prepared for histological studies and preserved at −80°C for either Western blot, lipid content evaluation, or RT-qPCR studies.

### 2.3 MMP activity

The gelatinase activities of MMP9 and MMP2 were studied in frozen preserved maternal plasma from control and GDM patients treated or not with the EVOO-enriched diet, and evaluated by zymography as previously described (26). Briefly, from maternal plasma, an amount equivalent to 30 µg of protein was mixed with loading buffer (2% SDS, 10% glycerol, 0.1% bromophenol blue, and 50 mM Tris-HCl, pH 6.8) and subjected to a 7.5% SDS-PAGE containing 1 mg/ml gelatin (type A from porcine skin). Following electrophoresis, gels were rinsed in 30% Triton X-100 for 60 min to remove SDS. Gels were incubated on 50 mM Tris Buffer, pH 7.4, containing 0.15 mM NaCl and 30 mM CaCl_2_ for 18 h at 37°C. Gels were stained with Coomassie blue and destained with 10% acetic acid–30% methanol in water. The areas of proteolytic activity appeared as negatively stained bands in a dark background. The identities of MMPs were based on their molecular weights and a positive internal control (conditioned medium of human fibrosarcoma HT-1080 cells). The enzymatic activity was quantified using ImageJ software and expressed as arbitrary densitometric units, which were normalized to the internal control. Data are shown as relative to a value of 1 assigned to the mean value for MMP activity in the control group.

### 2.4 Western blot analysis

FABP4 levels were evaluated in frozen preserved maternal plasma samples from the three experimental groups by Western blot. Proteins from maternal plasma (60 µg of protein) were separated by SDS-PAGE and transferred to nitrocellulose membranes (35V constant, overnight at 4°C), as previously described (42). The membranes were stained with Ponceau Red staining solution for total proteins (Sigma-Aldrich) to confirm proper loading and transfer. Blocking was carried out in 1% BSA in TBS-Tween solution for 1 h at room temperature. Next, the membranes were incubated with the primary antibody, diluted in 2% BSA in TBS-Tween, overnight at 4°C. A primary antibody from Genetex (CA, USA), diluted 1/500, was used to determine the protein expression of FABP4. A primary antibody from Sigma-Aldrich, diluted 1/500, was used to determine the protein expression of actin. After washing, the membranes were incubated with the appropriate peroxidase-conjugated anti-rabbit secondary antibody. The bands were visualized using ECL detection solution (Thermo Scientific, MA, USA) and captured in a Chemiluminescence imaging system (GeneGnomeXRQ, Syngene). Densitometry analysis was performed with ImageJ software. The relative intensity of protein signals was quantified by densitometric analysis using the ImageJ Software (NIH, MD, USA). Results are expressed as protein of interest/actin protein ratio.

### 2.5 Histochemical studies

Placental slides were paraffinized and serially sectioned (5 μm). The slides obtained were deparaffinized with xylol and rehydrated through a graded series of ethanol. To perform periodic acid–Schiff staining (PAS), a histological technique that allows the evaluation of glycogen depots (32), the slides were incubated with 0.5% periodic acid solution for 5 min, washed with distilled water, and stained with Schiff’s reagent, protected from light, for 15 min.

To perform Masson’s trichrome staining, a histological technique in which light blue color indicates collagen deposition (31), the slides were incubated with Weigert’s hematoxylin for 7 min, and then washed for 5 min. Subsequently, the slides were stained in scarlet-acid fuchsin for 2 min, rinsed in distilled water, incubated in phosphomolybdic acid for 15 min, stained with Light Green 2% for 10 min, and incubated in 1% acetic acid for 1 min.

To perform immunohistochemical studies, the slides were incubated for 20 min at 95°C to unmask the antigens. Next, slides were incubated with H_2_O_2_ 0.3% for 20 min to block the activity of the endogenous peroxidase. The sections were then incubated overnight with the primary antibody (anti-megalin mouse antibody, Santa Cruz Biotechnology, diluted 1/40; anti-Cubilin rabbit antibody, Millipore, diluted 1/20) at room temperature. Then, slides were incubated with the corresponding secondary antibody (Vector Laboratories, CA, USA, diluted 1/200) for 1 h and, after rinsing, incubated with the avidin–biotin complex (Vectastain, Vector Laboratories) for 1 h. The stain was developed with 3,3′-diaminobenzidine, as previously described (22). Negative controls were performed in the absence of primary antibody and by replacing the primary antibody by a pooled serum of the same species that contains a spectrum of the IgG subclasses (Vector Laboratories). In all cases, the tissues were dehydrated and mounted, and examined by two skilled blinded observers using light microscopy (Nikon Eclipse E200) and photographed (Nikon DS-Fi1). Immunoreactivity intensity was quantified using the ImageProPlus software. Data are shown as relative to a value of 1, assigned to the mean value of the control group.

### 2.6 Lipid content and fatty acid percentual composition

Placentas were each homogenized in 1,000 μl of PBS and protein content in the homogenates measured by the Bradford assay. As previously carried out (43), tissue lipids were extracted from 500 μl of each homogenate by three rounds of organic extraction in methanol:chloroform (2:1), following the method of Bligh and Dyer. The lipids extracted (equivalent to 400 μg of protein) were developed by thin-layer chromatography in 0.2- mm silica gel plates (Merck, Darmstadt, Germany). The developing solvent mixture was hexane:ether:acetic acid (80:20:2, v:v:v). Lipid species were stained with iodine vapors, identified and quantified by comparison with known amounts of standards on the same plate, and analyzed densitometrically with the ImageJ software.

Fatty acid methyl esters (FAMEs) extracted from placental lipids were prepared by reaction with 5% HCl in methanol at 70°C for 2 h. After cooling, water was added, and FAMEs were extracted with chloroform. FAMEs were analyzed by gas chromatography, as previously mentioned (43) on a Focus gas chromatograph (Thermo Finnigan Corporation), equipped with an Innowax capillary column (Agilent, 100% polyethylene glycol, 30 m length, 0.25 mm i.d., and 0.5 µm film thickness). Nitrogen was the carrier gas (0.8 ml/min continuous flow rate). The injector and detector temperatures were set at 240°C and 300°C, respectively. Column temperature was programmed from 100°C (1 min), then at a rate of 15°C/min up to 200°C, maintained at this temperature for 1 min, then at a rate of 2°C/min up to 240°C and maintained at 240°C for 17 min. Individual FAMEs were identified by comparing retention time data with those obtained from authentic laboratory standards (Sigma-Aldrich Co.). Quantitation was done by comparing the percent of area of each FAME peak on the chromatogram with that of the internal standard of known weight (nonadecanoate methyl ester, Sigma-Aldrich Co.). Individual FAMEs were expressed as percentage of total fatty acids.

### 2.7 qRT-PCR assay for mRNA and miR-199

Total RNA and microRNA were isolated from 100 mg of placental explants using RNAzol^®^ (MCR Inc., OH, USA) according to the manufacturer’s recommendations. The concentrations of total RNA and microRNA were determined using the NanoDrop spectrophotometer. For mRNA analysis, cDNA was synthesized incubating 2 μl (1 μg/μl) of extracted RNA and random primer hexamers (Promega, WI, USA) at 72°C for 5 min. Then, we added a reaction mixture containing MMLV reverse transcriptase (Promega) and each of all four dNTPs (Invitrogen) and performed an incubation at 37°C for 60 min and then at 72°C for 15 min, as previously mentioned (44). cDNA was used to perform the amplification in reaction buffer containing dNTPs mix (Solis BioDyne), GoTaq Polymerase (Promega), Eva Green 20× (Biotium, CA, USA), and the gene-specific primers described in **Table 1**. The qPCR started with a denaturation step at 95°C for 5 min and followed by up to 40 cycles of denaturation, annealing, and primer extension. mRNA levels were normalized to the geometric mean of the mRNA levels of the 60s ribosomal protein L30 and β2-microglobulin.

**Table 1:** Primer sequences of the primers used for qRT-PCR.

Gene	Primer Sequences	
	Forward	Reverse
***LDLR***	5′-GCTCTGTCCATTGTCCTCCC-3′	5′-TAGCTGTAGCCGTCCTGGTT-3′
***CLTC***	5′-TTTGTTTTGCAGTTCGGGGC-3′	5′-GGTTCCCTGTAGGTGGTGTG-3′
***LRP1***	5′-ATGGAGATCCGAGGTGTGGA-3′	5′-AGCACTGTGACGTTGTCGAT-3′
***LRP2***	5′-AGCCTCAACTGGGTTTTTGT-3′	5′-GTACACATTTAGCCACAGGGC-3′
***RPL30***	5′-TGATCAGACAAGGCAAAGCG-3′	5′-GCCACTGTAGTGATGGACACC-3′
***B2M***	5´-TGGGAAGAGGAGACACGGAA-3´	5´-GGCATACTGTTCATACCCGC-3´

The evaluation of miR-199 was performed as previously mentioned (44). Briefly, cDNA was obtained using the TaqMan MicroRNA reverse transcription kit (Applied Biosystems, CA, USA). The relative expression of miR-199 was determined using the TaqMan detection system (assay ID 000498, Applied Biosystems). U6 spliceosomal RNA (assay ID 001973, Applied Biosystems) was used as endogenous control.

For both mRNA and microRNA, the course of PCR amplification was followed in each cycle by the fluorescence measurement on Corbett Rotor-Gene 6000 (QIAGEN, MD, USA). The expression of the mRNAs evaluated and miR-199 was quantified using the 2^−ΔΔCt^ method. Relative mRNA levels are shown as fold values of the control.

### 2.8 Statistical analysis

Data are presented as means ± SEM. Homogeneity of variance was verified with Levene’s test and normality of the variable distribution was checked with the Shapiro–Wilk test using the SPSS 19 software. Once the ANOVA assumptions were verified, the experimental groups were compared by one-way ANOVA followed by Bonferroni’s *post-hoc* test, using the GraphPad Prism Version 8 software (GraphPad, Inc., San Diego, CA, USA). A *p-*value lower than 0.05 was considered statistically significant.

## 3 Results

### 3.1 Effect of the EVOO-enriched diet on markers of insulin resistance and proinflammation in maternal plasma of GDM women

To examine the putative beneficial effects of an EVOO-enriched diet supplementation on insulin resistance markers in GDM women, TG/HDL cholesterol ratio and FABP4 levels were evaluated in plasma samples in a cohort of control (*n* = 15), GDM (*n* = 15), and GDM patients who received the dietary EVOO supplementation from enrollment to term (*n* = 15).

**Figure 1:**
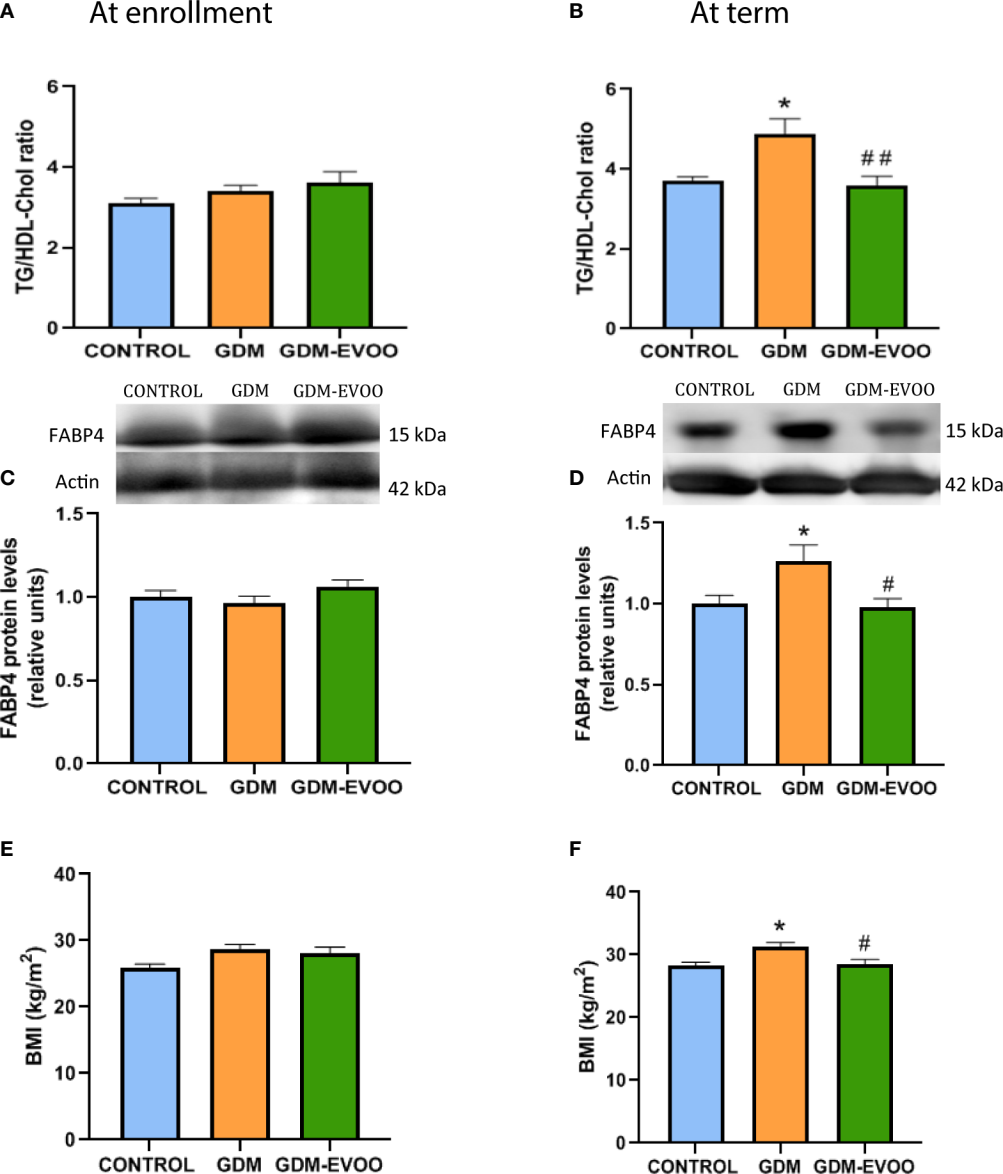
TG/HDL cholesterol ratio **(A)** at enrollment and **(B)** at term; FABP4 circulating levels **(C)** at enrollment and **(D)** at term; and maternal BMI **(E)** at enrollment and **(F)** at term in control pregnant women and GDM patients receiving or not receiving an EVOO-enriched diet from enrollment to term. Values represent mean ± SEM. Statistical analysis: One-way ANOVA in conjunction with Bonferroni’s test. *p < 0.05 vs. Control group, ^#^ *p* < 0.05 vs. GDM group, ^##^ *p* < 0.01 vs. GDM group.

At enrollment, the TG/HDL cholesterol ratio was unchanged in the evaluated experimental groups (**Figure 1A**). Differently, at term, the TG/HDL cholesterol ratio was increased in the GDM group compared to controls (*p* < 0.05), an alteration prevented in the GDM women who received the EVOO-enriched diet (*p* < 0.01, GDM-EVOO vs. GDM) (**Figure 1B**). At enrollment, FABP4 levels were unchanged in the experimental groups evaluated (**Figure 1C**). Differently, at term, FABP4 levels were increased in the GDM group compared to controls (*p* < 0.05), an alteration prevented in the GDM women who received the EVOO-enriched diet (*p* < 0.05 GDM-EVOO vs. GDM) (**Figure 1D**). BMI was also unchanged at enrollment in the three experimental groups (**Figure 1E**), whereas at term, it was increased in the GDM group compared to controls (*p* < 0.05), an alteration prevented by the EVOO-enriched diet (*p* < 0.05 GDM-EVOO vs. GDM) (**Figure 1F**).

As previous studies have shown increases in MMP9 in term placentas and in MMP2 and MMP9 in cord blood from GDM patients (22), the activities of MMP2 and MMP9 were evaluated in maternal plasma in the same cohort of control and GDM patients treated or not with the dietary EVOO supplementation.

**Figure 2:**
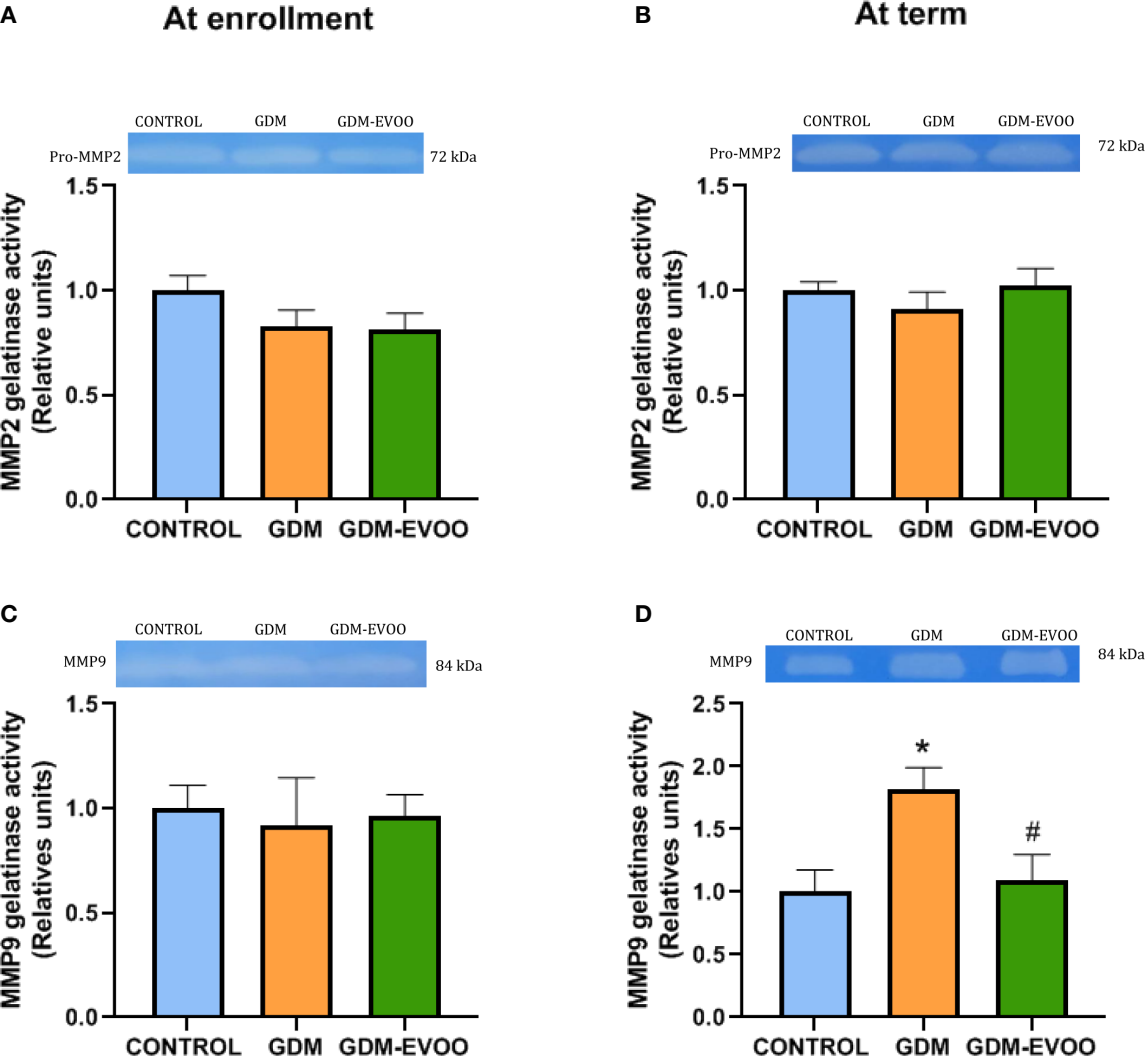
Plasma MMP2 gelatinase activity **(A)** at enrollment and **(B)** at term; plasma MMP9 gelatinase activity **(C)** at enrollment and **(D)** at term in control pregnant women and GDM patients receiving or not receiving an EVOO-enriched diet from enrollment to term. Values represent mean ± SEM. Statistical analysis: One-way ANOVA in conjunction with Bonferroni’s test. *p < 0.05 vs. Control group, ^#^ *p* < 0.05 vs. GDM group.

Maternal plasma MMP2 activity was unchanged in the experimental groups both at enrollment and at term (**Figures 2A, B**). Differently, MMP9 activity was unchanged in the maternal plasma of the experimental groups at enrollment but increased in the GDM group at term compared to controls (*p* < 0.05, **Figure 2C**). The increase in maternal plasma MMP9 activity in the GDM group was prevented by the maternal diet enriched in EVOO (*p* < 0.05 vs. GDM) (**Figure 2D**).

### 3.2 Effect of the EVOO-enriched diet on morphometric parameters and collagen, glycogen, and lipid content in the placenta of GDM women

The placenta is a crucial organ for the mother, the fetus, and the offspring’s health (6, 45). Previously, we found increased proinflammatory markers in the placenta from GDM patients, an alteration prevented by the maternal diet enriched in EVOO (22). Here, we focused on placental morphometric parameters and the evaluation of profibrotic markers and metabolic substrates that are stored in the placenta. As shown in **Table 2**, the diameter and perimeter of the placenta showed no differences in the groups evaluated. Differently, the thickness of the placenta was increased in the GDM group compared to controls (*p* < 0.05), an alteration prevented in the placenta of GDM women who received the EVOO-enriched diet (*p* < 0.01 GDM-EVOO vs. GDM) (**Table 2**).

**Table 2:** Placental morphometric parameters.

	Control (*n* = 15)	GDM (*n* = 15)	GDM-EVOO (*n* = 15)
Diameter (cm)	19.4 ± 0.5	18.6 ± 0.3	20.2 ± 0.7
Thickness (cm)	2.04 ± 0.09*	2.4 ± 0.07*	1.9 ± 0.12^##^
Perimeter (cm)	60.6 ± 1.0	57.8 ± 1.3	62.3 ± 2.1

Values represent mean ± SEM. Statistical analysis: One-way ANOVA in conjunction with Bonferroni’ test. *p < 0.05 vs. Control, ^##^p < 0.01 vs. GDM.

**Figure 3:**
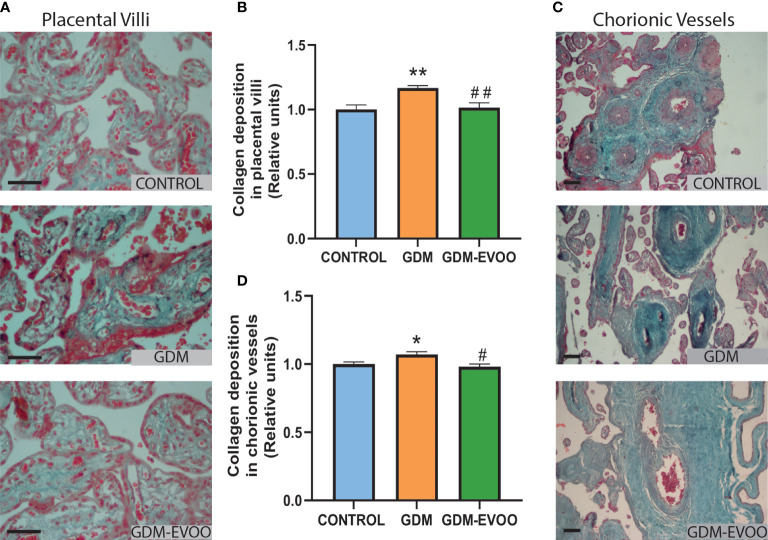
Collagen content evaluated by Masson’s trichrome staining in term placentas from control women and GDM patients receiving or not receiving an EVOO-enriched diet from enrollment and until term. **(A)** Representative Masson’s trichromic staining images and **(B)** densitometric analysis of collagen deposition in term placental villi; **(C)** representative Masson’s trichromic staining images and **(D)** densitometric analysis of collagen deposition in placental chorionic vessels. Collagen deposition is evident in the light blue-colored tissue. The black scale bars correspond to 50 μm (400×, placental villi) and 100 μm (200×, chorionic vessels). Values represent mean ± SEM. Statistical analysis: One-way ANOVA in conjunction with Bonferroni’s test. *p < 0.05 vs. Control group, **p < 0.01 vs. Control group, ^#^ *p* < 0.05 vs. GDM group, ^##^ *p* < 0.01 vs. GDM group.

Aiming to address the placental content of collagen, an abundant extracellular matrix protein, relevant for placental structure, development, and function and marker of fibrosis when increased (31, 46), we performed a trichrome Masson staining in term placentas from control women and from GDM patients who received or did not receive the EVOO dietary supplementation. We found increased collagen depots in the placental villi of GDM patients compared to controls (*p* < 0.01), an alteration prevented by the maternal diet supplemented with EVOO (*p* < 0.01 GDM-EVOO vs. GDM) (**Figures 3A, B** **)**. In addition, chorionic vessels also showed increased collagen depots in the placenta from GDM patients compared to controls (*p* < 0.05), an alteration prevented by the maternal diet supplemented with EVOO (*p* < 0.05 GDM-EVOO vs. GDM) (**Figures 3C, D**).

**Figure 4:**
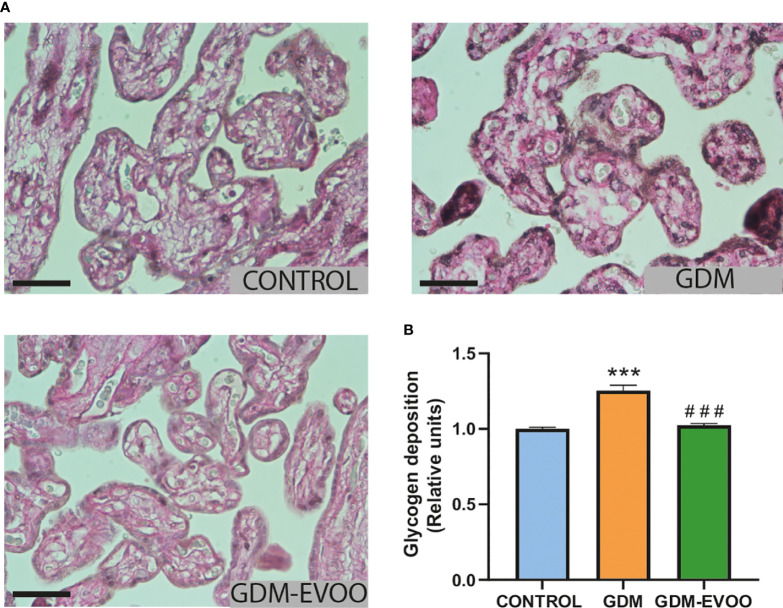
Glycogen content evaluated by PAS staining in term placentas from control women and GDM patients receiving or not receiving an EVOO-enriched diet from enrollment to term. **(A)** Representative PAS staining images and **(B)** densitometric analysis of PAS staining in term placental villi. PAS positive staining is evident in the magenta-colored tissue. The black scale bars correspond to 50 μm (400×). Values represent mean ± SEM. Statistical analysis: One-way ANOVA in conjunction with Bonferroni’s test. ***p < 0.001 vs. Control group, ^###^ *p* < 0.001 vs. GDM group.

**Figure 5:**
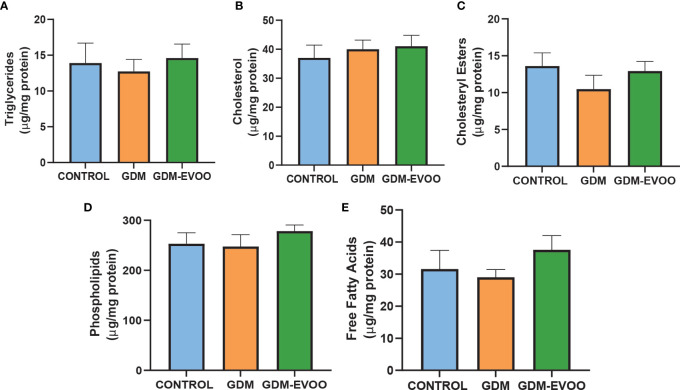
Lipid content in term placentas from control women and GDM patients receiving or not receiving an EVOO-enriched diet from enrollment to term. **(A)** Triglycerides, **(B)** cholesterol, **(C)** cholesteryl esters, **(D)** phospholipids, and **(E)** free fatty acids. Values represent mean ± SEM. Statistical analysis: One-way ANOVA in conjunction with Bonferroni’s test.

Next, we addressed the placental levels of glycogen, the main storage form of carbohydrates in different tissues, which can be evaluated by PAS histochemical staining (47). Increased glycogen depots, as indicated by increased PAS staining, was observed in the placenta from GDM patients compared to controls (*p* < 0.001), an alteration prevented by the maternal diet supplemented with EVOO (*p* < 0.001 GDM-EVOO vs. GDM) (**Figures 4A, B** **)**. On the other hand, the evaluation of term placenta lipid content showed no differences in triglycerides, cholesterol, cholesteryl esters, phospholipids, and free fatty acid levels when the three experimental groups were compared (**Figure 5** **)**. Similarly, no changes in the fatty acid percentual composition were observed when the three experimental groups were compared (**Table 3**).

**Table 3:** Percentage composition of fatty acids.

Fatty Acid	Control (*n* = 15)	GDM (*n* = 15)	GDM-EVOO (*n* = 15)
C16:0 palmitic acid	30.5 ± 1.1	27.8 ± 0.8	27.7 ± 0.6
C16:1 palmitoleic acid (n-7)	0.6 ± 0.08	0.7 ± 0.06	0.8 ± 0.07
C18:0 stearic acid	13.4 ± 0.3	14.2 ± 0.7	13.6 ± 0.6
C18:1 oleic acid (n-9)	11.8 ± 0.4	11.8 ± 0.4	12.0 ± 0.3
C18:2 linoleic acid (n-6)	14.9 ± 0.6	14.2 ± 0.7	14.6 ± 0.4
C18:3 α-linolenic acid	0.5 ± 0.02	0.5 ± 0.02	0.5 ± 0.03
C18:3 γ-linolenic acid	0.3 ± 0.02	0.7 ± 0.2	0.7 ± 0.1
C20:0 arachidic acid	0.15 ± 0.01	0.16 ± 0.01	0.16 ± 0.01
C20:1 gondoic acid (n-9)	0.3 ± 0.02	0.3 ± 0.02	0.3 ± 0.01
C20:3 eicosatrienoic acid (n-3/n-6)	4.1 ± 0.1	4.2 ± 0.2	4.0 ± 0.2
C20:4 arachidonic acid (n-6)	19.3 ± 0.6	21.0 ± 0.5	21.2 ± 0.7
C20:5 eicosapentaenoic acid (n-3)	0.4 ± 0.03	0.5 ± 0.04	0.5 ± 0.04
C22:4 docosatetraenoic acid (n-6)	0.7 ± 0.1	0.9 ± 0.1	0.9 ± 0.1
C22:5 docosapentaenoic acid (n-3/n-6)	0.7 ± 0.09	0.8 ± 0.08	0.8 ± 0.06
C22:6 docosahexaenoic acid (n-3)	1.5 ± 0.2	1.6 ± 0.1	1.7 ± 0.1

Values represent mean ± SEM. Statistical analysis: One-way ANOVA in conjunction with Bonferroni’s test.

### 3.3 Effect of the EVOO-enriched diet on molecules involved in the endocytic pathway in the placenta of GDM women

**Figure 6:**
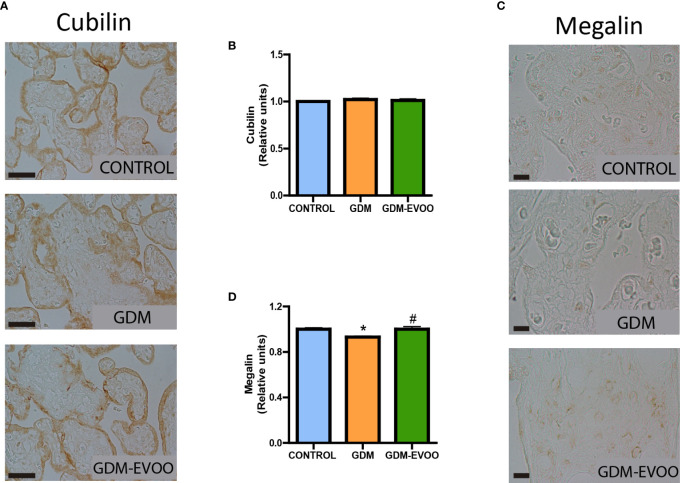
Cubilin and megalin levels evaluated by immunohistochemistry in term placentas from control and GDM patients receiving or not receiving an EVOO-enriched diet from enrollment to term. **(A)** Representative images of cubilin immunodetection and **(B)** densitometric analysis of cubilin in term placental villi; **(C)** representative images of megalin immunodetection and **(D)** densitometric analysis of megalin in term placental villi. The black scale bars correspond to 50 μm (400×, Cubilin) and 10 μm (1000×, Megalin). Values represent mean ± SEM. Statistical analysis: One-way ANOVA in conjunction with Bonferroni’s test. *p < 0.05 vs. Control group, ^#^ *p* < 0.05 vs. GDM group.

Although we found no changes in lipid content in the placenta, we previously found increased circulating triglycerides in the GDM patients, an alteration prevented by the EVOO-enriched diet (22). As lipoproteins rich in triglycerides may be transferred from maternal circulation to the placenta through the endocytic pathway (33), we evaluated the levels of cubilin and megalin, two proteins involved in the endocytosis of lipoproteins (48). The cubilin levels in term placentas showed no differences when the three experimental groups were compared (**Figures 6A, B**). Differently, megalin levels were reduced in the placenta from GDM patients compared to controls (*p* < 0.05), an alteration prevented by the maternal diet supplemented with EVOO (*p* < 0.05 GDM-EVOO vs. GDM) (**Figures 6C, D**).

**Figure 7:**
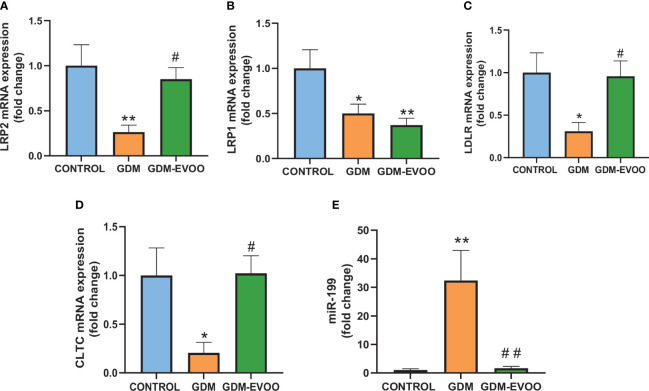
mRNA of genes involved in the endocytic pathway and miR-199 levels in the placenta of control women and GDM patients receiving or not receiving an EVOO-enriched diet from enrollment to term. **(A)** LRP2, **(B)** LRP1, **(C)** LDLR, **(D)** CLTC, and **(E)** miR-199. Values represent mean ± SEM. Statistical analysis: One-way ANOVA in conjunction with Bonferroni’s test. *p < 0.05 vs. Control group, **p < 0.01 vs. Control group, ^#^ *p* < 0.05 vs. GDM group, ^##^ *p* < 0.01 vs. GDM group.

The mRNA expression of LRP2 (which codifies for megalin), as well as the mRNA of other genes involved in the endocytic pathway—LRP1, LDLR, and CLTC, was further evaluated. We found reduced expression of LRP2, LRP1, LDLR, and CLTC in the placenta from GDM patients compared to controls (*p* < 0.05) (**Figures 7A–D**). The reduced mRNA levels of LRP2, LDLR, and CLTC were prevented by the EVOO-enriched diet (*p* < 0.05) (**Figures 7A, C, D**). As these genes are targets of the miR-199 (37), we evaluated its expression. Interestingly, miR-199 showed a 30-fold increase in its expression in the placentas of GDM patients compared to controls (*p* < 0.01), and alteration prevented in the placentas of the GDM patients treated with the EVOO-enriched diet (*p* < 0.01 GDM-EVOO vs. GDM) (**Figure 7E**).

## 4 Discussion

In this work, performed as a secondary analysis in a cohort of GDM patients who received or did not receive an EVOO-enriched diet, we provided evidence of the capacity of an EVOO-enriched diet to reduce maternal circulating markers of insulin resistance and the proinflammatory state. Moreover, in the placenta, morphological alterations, markers of fibrosis and glycogen accumulation, and impairments in proteins, genes, and miRs related to the endocytic pathway were prevented by the maternal diet enriched in EVOO. Therefore, this study points to the placenta, a crucial organ in the determination of the health of the mother and the offspring, as a target organ that highly benefits from the maternal dietary treatment with EVOO.

In this study, the GDM cohort evaluated consisted of women with good metabolic control, and without obesity, as obesity was an exclusion criterion (22). Most of the patients studied (83% (25/30) were diagnosed by the p75 glucose oral glucose test [2-h post-test values: GDM group 152.6 ± 4.2 (*n* = 12); GDM-EVOO group 152.4 ± 2.4 (*n* = 13), values significantly higher than those observed in the Control group 98.4 ± 6.2 mg/dl (*n* = 15), *p* < 0.001 vs. GDM and GDM-EVOO groups]. Although metabolic control was good in both GDM and GDM-EVOO patients, GDM is a complex pathology characterized by changes in glucose and lipid metabolism and by a prooxidant and proinflammatory intrauterine environment, which possibly leads to adverse effects even when metabolic control is achieved (9, 11). It is known that there is a close relationship between the intrauterine proinflammatory state and maternal insulin resistance (9). In this work, GDM women showed no changes in the insulin resistance markers evaluated or BMI compared to controls at enrollment. Differently, at term, the TG/HDL cholesterol ratio and FABP4 circulating levels were increased in GDM patients receiving the standard recommended diet for pregnancy compared to controls. The prevention of the altered insulin resistance markers observed in the GDM patients treated with the EVOO-enriched diet suggests that the EVOO dietary treatment is able to regulate insulin resistance, likely related to the concomitant reduction observed in maternal BMI. Although there are no previous studies addressing the effect of EVOO on insulin resistance markers in GDM, different studies performed out of pregnancy have shown the capacity of an EVOO-enriched diet to reduce parameters related to insulin resistance (12, 49). Of note, as stated in our primary study, the nutritional indication to the GDM-EVOO group (3 tablespoons/day) was evaluated by nutritionists at least monthly, and good adherence (over 26 g EVOO per day at least 5 days a week) was reported in 83% of the patients (22). Differently, in the control and GDM groups, there was almost no consumption of EVOO.

Increased insulin resistance in GDM is associated with a proinflammatory state (9, 10). The gelatinolytic overactivity of MMP9 has been related to a proinflammatory state in different pathologies (25, 50). Furthermore, previous studies have shown that diets enriched in EVOO prevent MMP overactivity in maternal plasma, placentas, and fetuses in experimental models of diabetes and pregnancy (26). This capacity to reduce proinflammatory markers in the maternal, placental, and fetal compartments seems to occur also in GDM patients. Indeed, in this study, we found increased activity of MMP9 in maternal plasma in the GDM group, an alteration prevented by the maternal diet enriched in EVOO, whereas in our primary study performed in this cohort of GDM patients, we found that the EVOO-enriched diet prevents MMP gelatinolytic overactivity in the placenta and cord blood of GDM patients (22).

Our primary study showed no changes in placental and fetal weight when control, GDM patients, and GDM patients treated with the EVOO-enriched diet were compared (22). Although macrosomia and larger placentas are common in GDM (2, 51), in the population evaluated, both the fact that obesity was excluded and the very good metabolic control of all the patients of the study are likely related to the normal average weight of the placentas and the neonates. In this work, we studied the placental morphology, and although the placental diameter and perimeter were not changed in the GDM patients treated or not with the EVOO-enriched diet compared to controls, increased thickness of the GDM placentas was observed, an alteration that may be related to impaired placental function and/or fetal programming, as suggested by studies performed in different pathologies (6, 52). The fact that placental thickness was increased in the GDM group despite the similar perimeter and weight, alterations prevented by the EVOO-enriched diet, may be related to alterations in the extracellular matrix remodeling, which may change the shape of the placenta. In this regard, we have previously found increases in placental MMP9 activity, an alteration prevented by the EVOO-enriched diet (22), and, in this study, we addressed extracellular matrix components and storage metabolites possibly involved in the change observed in the placental thickness.

We first evaluated collagen content, a marker of fibrosis. Collagen content was increased not only in placental villi but also in chorionic vessels in GDM placentas, an alteration that may contribute to placental thickness and impair maternal–fetal exchange (31) and that was prevented by the maternal diet enriched in EVOO. Out of pregnancy, experimentally induced liver fibrosis has been found to be prevented by a diet enriched in EVOO (53).

We next evaluated placental glycogen depots and lipid accumulation as putative contributors to placental thickness. PAS staining indicated increased glycogen deposition in the placentas from GDM patients compared to controls. Similarly, other studies have shown increased glycogen deposition in placentas from GDM patients, an alteration related to the hyperglycemic and proinflammatory environment (54, 55). Interestingly, we found that glycogen accumulation was prevented by the maternal diet enriched in EVOO in GDM patients, an effect that may reflect the prevention of increased insulin resistance and amelioration of the increased glucose transfer from the maternal circulation to the placenta.

On the other hand, the lack of changes in major lipid species levels and free fatty acid composition observed in placentas of the GDM women despite the context of increased insulin resistance led us to hypothesize a reduction in the incorporation of lipoproteins through the placental endocytic pathway. There were no changes in cubilin levels in the GDM placentas of patients treated or not with the EVOO-enriched diet, but the reduction in megalin levels observed in the GDM placentas suggested putative impairments in the incorporation of lipoproteins through the placenta. Although reduced megalin levels and increased megalin urine loss are clearly involved in diabetic nephropathy (56), as far as we know, this is the first report of altered levels of megalin in GDM placentas. In this work, the observed capacity of the EVOO-enriched diet to prevent the reduced levels of megalin prompted us to further evaluate the gene expression of other proteins involved in the endocytic pathway. Interestingly, the expression of LRP2, LRP1, CLTC, and LDLR was reduced in the GDM placentas compared to controls, suggesting that the placental endocytic pathway is profoundly affected by GDM. Although the relationship of the endocytic pathway with placental dysfunction still needs clarification (33), it is interesting to note that the reductions in LRP2, LDLR, and CLTC were prevented by the maternal diet enriched in EVOO. A well-known microRNA that has been found to negatively regulate the endocytic pathway is miR-199 (37). Notably, a 30-fold increase in miR-199 was observed in the placenta from GDM patients compared to control, an alteration prevented by the maternal diet enriched in EVOO. These results point to miR-199 as a putative dysregulator of the endocytic pathway in GDM placentas and to the EVOO-enriched diet as a nutrigenomic diet capable of regulating miR-199, overexpression although the implication of this new knowledge deserves further research.

Indeed, some of the parameters evaluated showed significant differences, although not huge, in the GDM group compared to the control group, suggesting that there are many small impairments in the GDM group, affecting different aspects of the maternal glucose and lipid metabolism and related to the placental metabolism and function. The fact that most of these impairments were prevented by the EVOO-enriched diet suggests that this could be a relevant nutritional treatment to improve the function of the placenta and the health of both the mother and the offspring. Future studies are needed to evaluate this in larger populations.

As limitations, we were not able to include a control group supplemented with EVOO and could not evaluate fatty acid residues in isolated syncytiotrophoblast plasma membranes, highly relevant for placental function and nutrient transfer and altered in different pathologies (57, 58).

In conclusion, the results of the present study provide evidence of benefits of an EVOO-enriched diet in GDM patients, due to its ability to regulate maternal insulin resistance and proinflammatory circulating markers, as well as to regulate collagen deposition, glycogen content and proteins, genes, and regulators of the endocytic pathway in the placenta. This secondary study sums to our primary study in a cohort of GDM patients treated with EVOO to altogether demonstrate the beneficial effects of this dietary treatment in GDM mothers and their placentas, effects that may ameliorate fetal development and may help prevent adverse fetal programming.

## Data availability statement

The raw data supporting the conclusions of this article will be made available by the authors, without undue reservation.

## Ethics statement

The studies involving human participants were reviewed and approved by Ethics and Research Committee of the Pirovano Hospital (Review Board Project: DI-2016-29-HGAIP). The patients/participants provided their written informed consent to participate in this study.

## Author contributions

Conceptualization: AJ. Study design: DGR, ED, MF, HG, EC, and AJ. Investigation: DGR, VC, and EC. Formal analysis and interpretation: DGR, VC, MM, EC, and AJ. Funding acquisition: AJ. Enrollment, follow-up, samples, and data collection: ED, MF, HG, SM, CG, and DGR. Writing—original draft: AJ. Writing—review and editing: DGR and EC. All authors contributed to the article and approved the submitted version.
